# *spalt* is functionally conserved in *Locusta* and *Drosophila* to promote wing growth

**DOI:** 10.1038/srep44393

**Published:** 2017-03-16

**Authors:** Dan Wang, Juanjuan Li, Suning Liu, Hang Zhou, Long Zhang, Wangpeng Shi, Jie Shen

**Affiliations:** 1 Department of Entomology, China Agricultural University, Beijing 100193, China

## Abstract

*Locusta* has strong fly wings to ensure its long distance migration, but the molecular mechanism that regulates the *Locusta* wing development is poorly understood. To address the developmental mechanism of the *Locusta* flying wing, we cloned the Dpp target gene *spalt (sal*) and analyzed its function in wing growth in the *Locusta*. The *Locusta* wing size is apparently reduced with vein defects when *sal* is interfered by injection of dsRNA, indicating that *sal* is required for locust wing growth and vein formation. This function is conserved during the *Drosophila* wing development. To better understand *sal*’s function in wing growth, we then used *Drosophila* wing disc as a model for further study. We found that *sal* promotes cell proliferation in the whole wing disc via positive regulation of a microRNA *bantam*. Our results firstly unravel *sal*’s function in the *Locusta* wing growth and confirm a highly conserved function of *sal* in *Locusta* and *Drosophila*.

*Locusta migratoria* is one of the global destructive migratory agricultural pests. Swarm formation and long-distance flight behavior are the two major reasons why *Locusta* plagues are so destructive even today^1^,^2^. During the migratory stage, these insects can keep flying for hours over hundreds of kilometers^2^. Therefore, besides gene expansion associated sufficient energy supply^3^, well developed and functional wings are required to adapt their migratory behavior^4^,^5^.

*Locusta* is an incomplete metamorphosis insect whose young nymph resembles the adult with visible developing forewings and hindwings^6^. The wing shape and size are varied among geographical populations. Based on forewing shape, grasshopper *Trilophidia annulata* can be divided into geographical groups^7^ and mircoRNAs are associated with the polymorphism of adult wings^8^. Biomechanical study shows that the fan-like distribution of veins improve fracture toughness in *Locusta*^9^,^10^. However, little is known about the molecular mechanism of how the *Locusta* wing develops into such delicate structure.

Current understanding of insect wing development mechanism is mainly from the fruitfly *Drosophila melanogaster*. In comparison, the fruitfly, a Diptera insect, undergoes complete metamorphosis. The adult wings are developed from larval wing imaginal discs which are formed from late embryogenesis and undergo intricate cell proliferation, differentiation and morphogenesis during larval and pupal stage inside the body. The pattern formation is delicately regulated by organizers located in the anterior/posterior (A/P) and dorsal/ventral (D/V) boundaries which secrete signal molecules including the long-range morphogens Decapentaplegic (Dpp) and Wingless (Wg)^11^,^12^, and short-range morphogen Hedgehog (Hh)^13^. These morphogens form gradients to regulate the expression of their target genes and control almost all aspects of wing development^12^.

The Dpp morphogen gradients controls cell growth through negatively regulation of the transcriptional suppressor *brinker (brk*), which downregulates *bantam (ban*), a developmentally regulated mircoRNA^14^,^15^,^16^. *ban* promotes cell proliferation autonomously and suppresses proliferation-induced apoptosis^17^. Various Dpp targets identified, such as *optomotor-blind (omb*), *spalt (sal*), and *vestigial (vg*), are repressed by *brk*^15^,^17^,^18^,^19^. Among them, *omb* represses *ban* expression in the medial regions of the wing disc to promote cell proliferation while plays an opposite role in the lateral regions^20^. The two *Drosophila sal* homologues *spalt major (salm*) and *spalt related (salr*) are also highly expressed in the central region of the wing pouch and mediate Dpp signaling pathway by promoting cell proliferation^21^,^22^. *salm* and *salr* have similar function but are not fully redundant^23^. For example, *salm*, but not *salr*, is evolutionally conserved in insects as a main regulator of fibrillar flight muscle fate^24^. Downstream the Salm/Salr complex, the vein-specific expression of the *knirps* and *iroquois* gene complexes and a serious of candidate genes are regulated for wing vein patterning and growth^25^,^26^. *sal* also participates in defining fly notum and wing hinge^27^ and the melanin pigmentation of wing eyespots in butterflies^28^,^29^,^30^. Therefore, *sal* may play a conserved role in insect wing development to generate the fast oscillating wings and whether *sal* functions through *ban* remains to be illustrated.

To better understand the developmental mechanism of *Locusta* flying wings, we cloned *Locusta sal* cDNAs and investigated the role of *sal* during wing blade and vein formation. By RNA interference (RNAi), we found that *sal* is required for wing growth and vein formation, which is conserved between the *Locusta* and *Drosophila*. Then, using the *Drosophila* wing disc model we dissected *sal* function in wing growth and found that *sal* promotes cell proliferation rate during larval wing development. Furthermore, mircoRNA *bantam* mediates the role of *sal* in cell proliferation regulation.

## Materials and Methods

### *Drosophila* strains and *Locusta*

All fruit flies used were *Drosophila melanogaster*. Flies were raised at 25 °C. Three transgenes *UAS-salm*^27^, *UAS-salmRNAi* (Tsinghua Fly Center THU3581) and *UAS-salrRNAi* (THU3147) were used to manipulate the expression level of *sal*. The efficiency of these RNAi lines has been recently verified by Tang *et al*.^31^. The Gal4 lines used were *ci-Gal4*^32^, *sal-Gal4*^33^, and *ap-Gal4*^34^ to drive the expression of the UAS transgene. The *bantam* enhancer reporter line was *br-C12-lacZ*^35^.

*Locusta migratoria* was raised at 28 °C with 60% relative humidity.

### Identification and sequence analysis of *spalt* from *L. migratoria* transcriptome

The available *salm* and *salr* gene sequences from *Drosophila* were used as references to screen *L. migratoria* transcriptome database we previously established^6^. The potential candidates of *L. migratoria sal* genes were confirmed by searching the BLASTX algorithm against the non-redundant NCBI nucleotide database using a cut-off E-value of 10^−5^.

Based on the *Drosophila* Spalt protein sequences, the deduced *Locusta* Spalt protein domains were determined by using DNAMAN software (Lynnon Biosoft). Other insect Spalt and Spalt-like proteins are obtained from NCBI (https://www.ncbi.nlm.nih.gov/protein). Global protein alignment and neighbor-joining phylogenic tree were performed by Geneious R9.

### RNA interference

The primers to make the double strand RNA probes were as follows:

Lmsal411-F 5′-ATGTTGCAGCGGCGTGCACAAGAGG-3′

Lmsal411-R 5′-TTGTCCCAGTTGTGCCAGCAGTGGA-3′

Lmsal468-F 5′-GCCATAGACCCTGCTAAGGACCCAG-3′

Lmsal468-R 5′-GTCCTTCACCTCTGCAGCTTGTATC-3′

GFP-F 5′-CACAAGTTCAGCGTGTCCG-3′

GFP-R 5′-GTTCACCTTGATGCCGTTC-3′

Lmsal411-dsRNA, Lmsal468-dsRNA, and GFP-dsRNA were injected into the 4^th^ instar nymphs using 20 μg dsRNAs per insect. Nymphs were raised at 28 °C for 10 days until they grew up to the 5^th^ instar. Imaging the whole body and wing discs using SONY DSC-HX1camera before the wing discs were lysed.

### Quantitative Real-Time RT-PCR Analysis

Total RNAs were extracted with Trizol reagent from the 5^th^ nymph wing discs, digested with DNase I, and reverse transcribed using the FastQuant cDNA Synthesis kit (Tiangen Biotech), then quantitative real-time RT-PCR was performed using Go Taq qPCR Master Mix (Promega). The primers for quantitative RT-PCR were as follows:

Lmsal411q-F 5′-GAGAATGCCAGCCAGGGTC-3′

Lmsal411q-R 5′-CGGTGCTTGAAGAAGGGTT-3′

Lmsal468q-F 5′-ACCAAAACATCGGAGACA-3′

Lmsal468q-R 5′-ATAGGACACGGTGGCAGA-3′

Relative transcript levels were assessed using the Comparative CT method. β-actin was used as an internal control.

### BrdU staining

BrdU staining was performed as previously described^20^,^36^. Dissected third instar wing discs were co-cultured with BrdU (1:100) in Schneider’s medium for 40–50 min at 25 °C. Then, samples were fixed in 4% formaldehyde and washed in PBT before immunostaining.

### Immunohistochemistry

The primary antibodies used were mouse anti-BrdU, 1:100 (MBL) and mouse anti-β-galactosidase1:2000 (Promega Z3783), and the secondary antibody was anti-mouse DyLight 549 (1:200, Agrisera). Images were obtained using an Olympus FV10-ASW laser scanning confocal microscope and processed with Adobe Photoshop 8.0.

### Wing size measurement

Image-J program was used to measure the wing areas and vein distances. High resolution images were opened in Image-J program, a straight line was drawn at the wanted sites between L2 and L4 veins to calculate the distance; and a wanted region as outlined along the L2 and L4 veins (for *Drosophila* wing) or the wing margin (for *Locusta* wing) to calculate the areas.

## Results and Discussion

### Identification of *Locusta sal* genes

**Figure 1:**
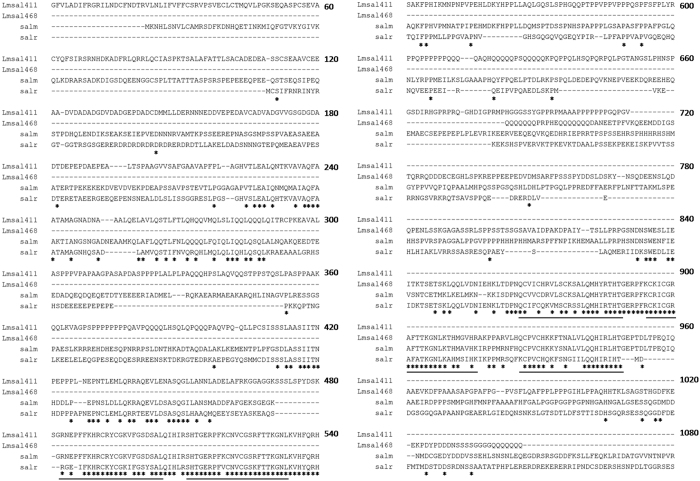
Identification of *sal* in *Locusta*. Two unigenes named *Lmsal411* and *Lmsal468* are found in locusts. Protein sequence alignment of *Locusta* and *Drosophila* Sal shows highly conserved sequences in the ZnF-C2H2 domains. Stars indicate the identical amino acids. Underlines show the putative *Drosophila* Salm ZnF-C2H2 domains.

**Figure 2:**
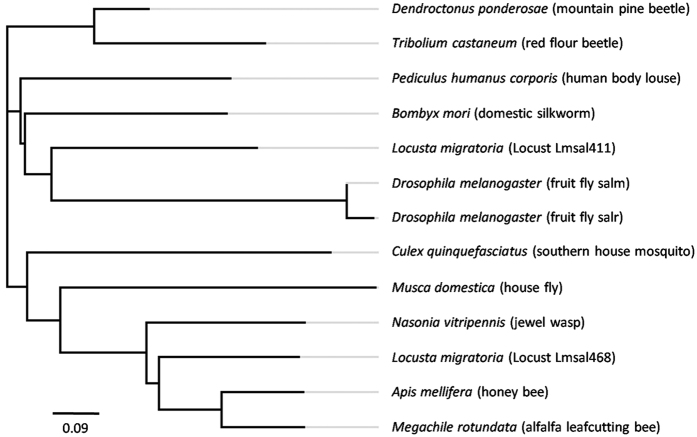
Phylogenic tree of *sal* in insects. The amino acid sequences of *L. migratoria* a12971 and a6922, and potential Sal from other insects are used to build the neighbor-joining tree.

Based on the transcriptome of the *Locusta* wing disc in our previous study^6^, we blasted the cDNA sequences with *salm* and *salr* in *Drosophila* and identified two orthologous unigenes a12971 and a6922, which are 2087 bp and 1073 bp, respectively, in the *Locusta* (Sup. Fig. 1). We named these two putative genes as *Lmsal411* and *Lmsal468*, respectively. Although these two unigenes were incomplete *spalt* gene sequences, both putative translations were predicted (Sup. Fig. 1). Protein sequence alignment showed that these two putative translations were 23.8% identity. Lmsal411, which was aligned with the anterior part of *Drosophila* Sal, was 33.1% identical to both Salm and Salr, while Lmsal468, aligned with the posterior part, showed a little higher identity (42.1% and 35.5%, respectively) (Fig. 1). The ZnF-C2H2 motifs showed a highly conservation in Lmsal411 (87.0% identity) and Lmsal468 (72.4% identity), indicating a conservative DNA binding motif of transcription factors. In addition, the phylogenetic analysis from different insect species revealed that Lmsal411 clustered with *Drosophila sal*, while Lmsal468 with bees (Fig. 2). Therefore, due to the less identity between these two translations and higher similarity to *Drosophila salm*, we speculate that these two unigenes may be different loci of the same *Locusta spalt*.

Above analysis implies that *Locusta sal* may share partial functions with that of *Drosophila*. In the *Drosophila* wing, the transcription factors Salm and Salr participate in wing growth^21^ and vein formation^18^,^25^. However, the regional growth effects of Sal on wing growth and cell proliferation rate have not yet intensively investigated.

### *Locusta sal* regulates wing growth

**Figure 3:**
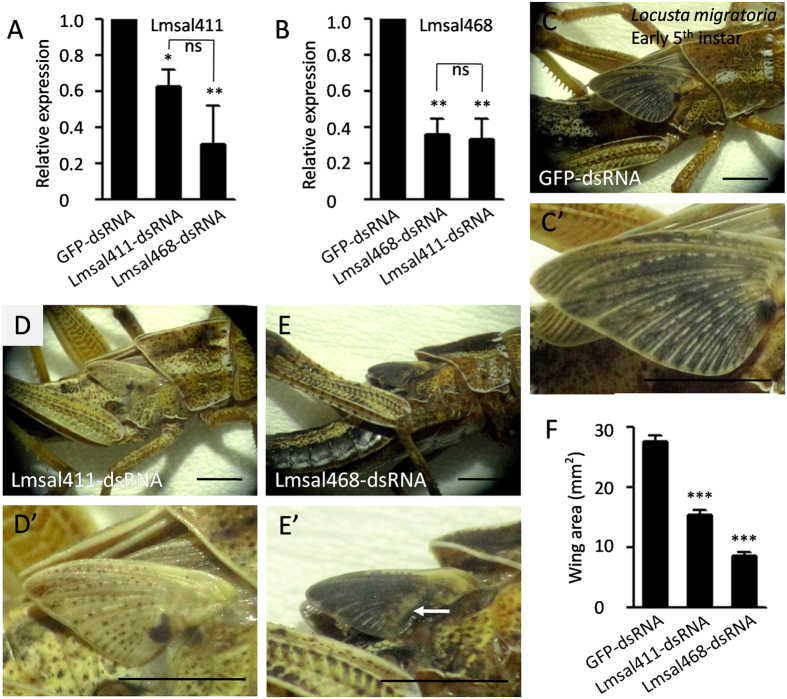
Knockdown of *salm* in *Locusta* leads to smaller wings. (**A** and **B**) Relative expression of the two genes in the wing discs of 5^th^ instar nymphs after *Lmsal411* dsRNAi (**A**) or *Lmsal468* dsRNAi (**B**) was injected into 4^th^ instar nymphs for 10 days. (**C**) The body and wing size are normal after GFP-dsRNA control injection. (**D**) The wing is smaller in Lmsal411-dsRNA injected locusts. (**E**) The wing is much smaller in Lmsal468-dsRNA injected locusts. (C’,D’and E’) show the higher magnification in (**C**,**D** and **E**), respectively. (**F**) Quantification of wing area. In each genotype, five nymphs were injected which give similar results as shown. *, ** and *** represent significant difference with the GFP-dsRNA control (pairwise comparison of t-tests, p < 0.05, 0.01, and 0.001, respectively). ns means no significant difference. Scale bar represents 4 mm.

To test the functional conservation of *sal*, we performed RNA interference experiments in *Locusta* using *Lmsal* genes we identified. After 10 days of injection, the mRNA levels of each gene were measured using quantitative real-time RT-PCR. The mRNA expression levels of *Lmsal411* and *Lmsal468* were significantly reduced to 62.7 ± 9.1% (*p* < 0.05) and 36.1 ± 8.5% (*p* < 0.01), respectively, when the corresponding gene was knocked-down (Fig. 3A and B). We noted that *Lmsal411* mRNA was reduced to 30.3 ± 21.8% when interfering with *Lmsal468*, and vise versa, *Lmsal468* mRNA was reduced to 33.5 ± 11.3% when interfering with another (Fig. 3A and B). Thus, the expression of these two unigenes are dependent on each other, implying that they belong to either the same gene or one gene complex. As when knocking-down either gene, the efficiency of interference of both genes were similar and had no statistic difference (Fig. 3A and B), and taken together with the above analysis of sequence identity, it is more likely that they are belonging to the same gene. When *Lmsal411* was interfered in 4^th^ instar, the wings of early 5^th^ instar nymphs were remarkably reduced to 45% of the control (15.4 vs 27.5 mm^2^) (Fig. 3D and F). Consistently, interfering with *Lmsal468* resulted in 69% loss of wing area (8.6 vs 27.5 mm^2^) (Fig. 3E and F). The phenotypes of *Lmsal468-RNAi* were much more obvious than that of *Lmsal411-RNAi* (Fig. 3D and E) probably due to the higher RNAi efficiency of the former. Moreover, the hindwing concomitantly reduced with the forewings, while the body size was not apparently affected (Fig. 3D and E). The veins in the posterior part was slightly affected in *Lmsal468-RNAi* nymphs (arrow in Fig. 3E’). This result indicates that *Locusta sal* regulates wing growth and vein pattern formation.

### *Drosophila sal* promotes adult wing growth

**Figure 4:**
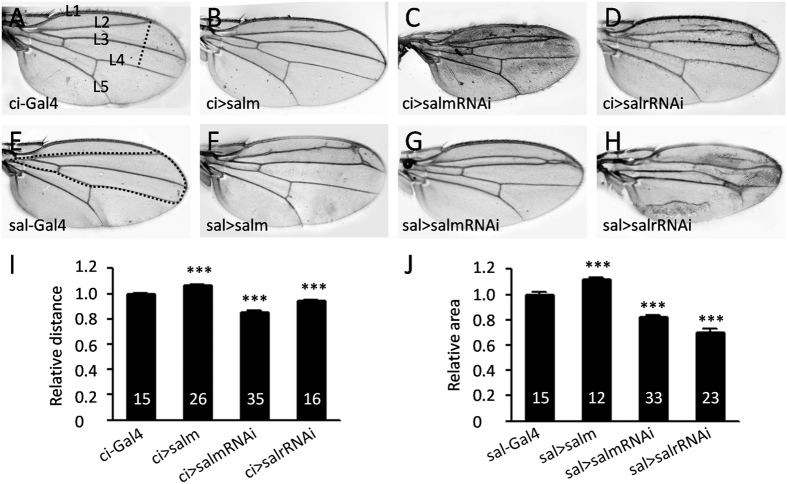
*sal* promotes wing growth in *Drosophila*. (**A**) Adult wing of *ci-Gal4* control shows the normal wing. L1‒L5 indicates the five longitudinal veins. The dashed line from the distal end of L2 crossing L3 and extending to L4 indicates the distance between L2 and L4. (**B**) The wing is larger in *salm* overexpressed flies. (**C** and **D**) Wings are smaller when *salm* or *salr* is knocked-down and the L2 and L3 veins are fused. (**E**) Adult wing of *sal-Gal4* control shows the normal wing. The dashed line circles the whole area between L2 and L4. (**F**) The anterior wing is larger in *salm* overexpressed flies. (**G** and **H**) Wings are smaller when *salm* or *salr* is knocked-down and the L2 and L3 veins are fused, similar with *ci-Gal4* driving wings. (**I**) Relative distance between L2 and L4 represented by dashed line in (**A**). (**J**) Relative areas circled by L2 and L4 represented by dashed line in (**E**). The number in (I) and (J) shows the quantification number. ***Represents significant difference (pairwise comparison of t-tests, p < 0.001).

To further confirm *sal* is functionally conserved in other insects to promote wing growth, we regionally up or down-regulated *salm* and *salr* in *Drosophila* wings and examined the adult wing size. *Drosophila* wing has five longitudinal veins, which are L1-L5 from the anterior to posterior part. Between L1 and L4 is the anterior part and other areas are the posterior wing. The distance from L2 to L4, which is indicated by a dashed line in Fig. 4A, was measured to monitor the size of the anterior part of the wing. The flies containing *ci-Gal4*, which is expressed in the anterior part of the wing, was used as a control and showed normal size and morphology. When *salm* was overexpressed, the relative distance was significantly increased (1.07 ± 0.01), while when knocked-down *salm* or *salr*, the distance was strikingly reduced (0.85 ± 0.01 and 0.94 ± 0.01, respectively) (Fig. 4B–D and I). To confirm this result, we used another driver *sal-Gal4*, which is expressed in the medial pouch to repeat the manipulation. Again, the relative area enclosed by L2 and L4 (sal-Gal4 expression region) was increased in *salm* overexpression wings and reduced in *salm* and *salr* knockdown wings (Fig. 4F–H and J). In addition, *sal* regulated the vein formation in all tested lines except *ci-Gal4*>*salm* which is consistent with previous studies^21^,^25^,^26^. These data confirm that *Drosophila sal* regulates wing growth and vein pattern formation. Thus, *sal* function in promoting wing growth is conserved in the fruitfly and locust.

### *sal* promotes cell proliferation in the wing disc

**Figure 5:**
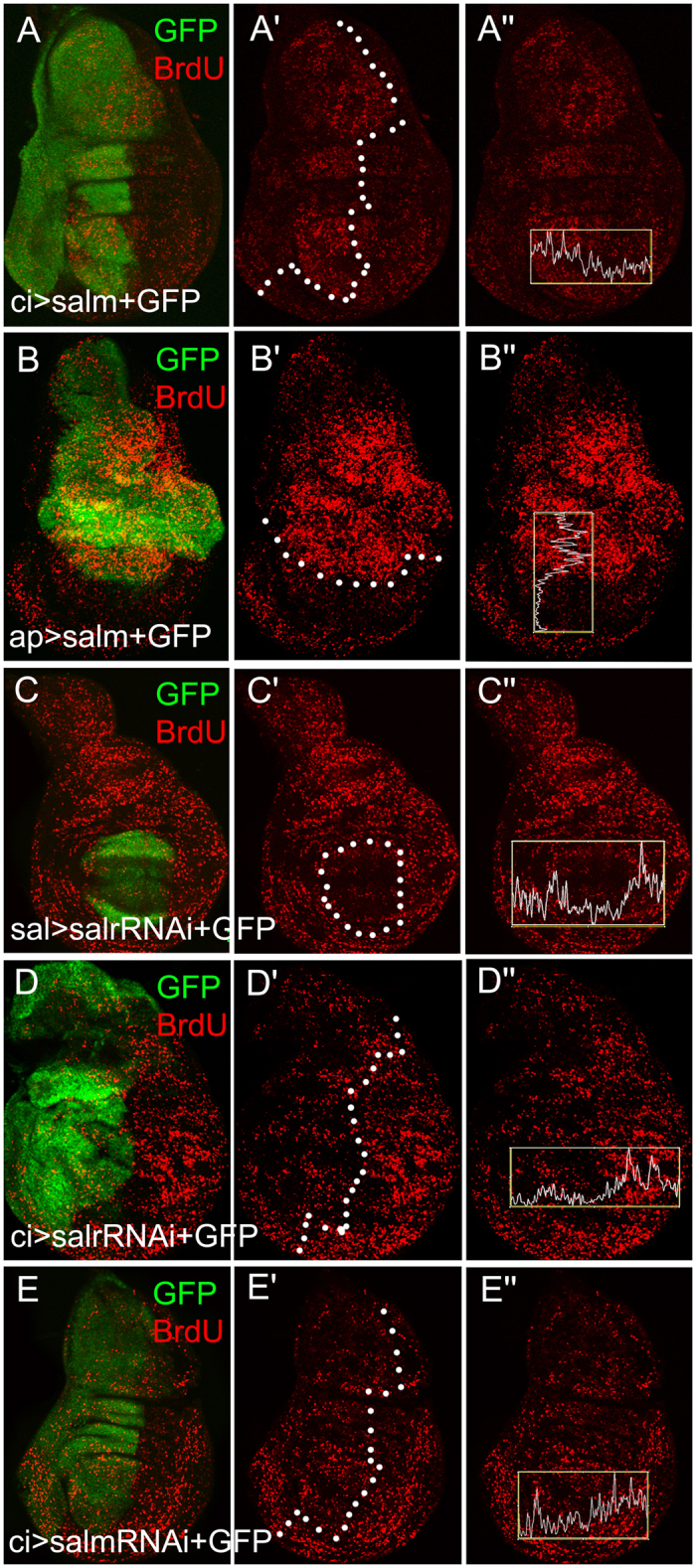
*sal* promotes cell proliferation in the *Drosophila* wing disc. (**A**) The cell proliferation rate is increased when *salm* is overexpressed in ci-Gal4 region. (**B**) The cell proliferation rate is increased when *salm* is overexpressed in ap-Gal4 region. (**C** and **D**) The cell proliferation rate is repressed when *salr* is knocked-down. (**E**) The cell proliferation rate is repressed when *salm* is knocked-down. GFP shows the Gal4 expressing domain. White boxes define the area of fluorescence quantification of BrdU staining.

To unravel the mechanism of wing growth regulated by *sal*, we use the *Drosophila* wing disc as the research model because it is easy of molecular labeling. We examined the cell proliferation rate in *Drosophila* wing discs by Bromodeoxyuridine (BrdU) incorporation which is widely used in cell proliferation studies. BrdU is a thymidine analog that can be incorporated into DNA during DNA synthesis in culture, and then be detected by specific anti-BrdU antibodies following with quantitative analysis of the anti-BrdU fluorescent intensities. In wild-type control, BrdU level is evenly distributed in the wing discs^20^. Elevating Salm level by expressing *UAS*-*salm* in the *ci-Gal4* or *ap-Gal4* domain, the level of BrdU level was increased (Fig. 5A and B). Within the pouch, the fluorescence intensity of BrdU was increased in the *salm* expressing regions, indicating an enhanced proliferation rate (Fig. 5A and B). Vice versa, compromising *salm* and *salr* by expressing *UAS*-*salm-RNAi* or *UAS*-*salr-RNAi* in the *sal-Gal4* or *ci-Gal4* domain, the level of BrdU staining was reduced which means that the cell proliferation rate was decreased. These data demonstrate that *Drosophila sal* promotes cell proliferation in the early wing developmental stages.

### *sal* promotes microRNA *bantam* expression

**Figure 6:**
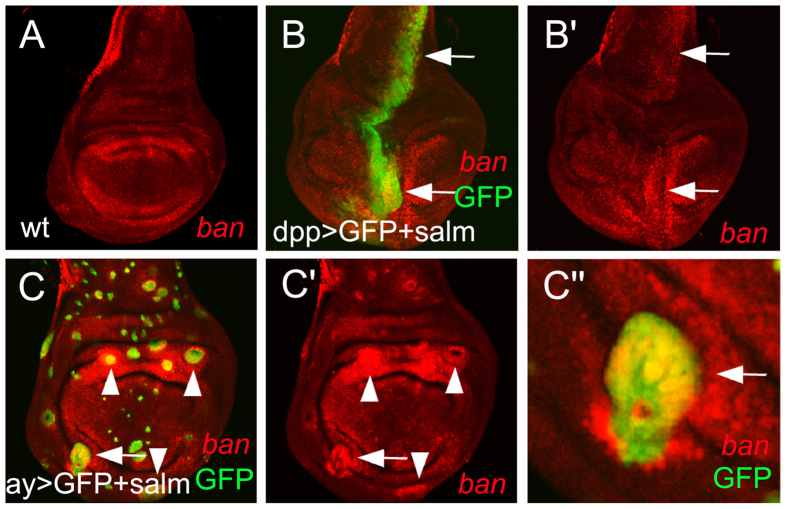
mircoRNA *bantam* is upregulated by *sal*. (**A**) The expression pattern of *br-C12-lacZ* reporter in the wild-type wing disc. (**B**) *ban* is elevated in *salm* overexpression cells (GFP positive) in both pouch and notum regions. (**C**) *salm* overexpression clones show upregulated *ban* level in the whole wing disc. (C”) is the magnification of the arrow-indicated clone in (**C**).

We previously found that one of the Dpp target genes *omb* represses *ban* expression in medial regions of the *Drosophila* wing discs while plays an opposite role in lateral regions^20^. Subsequently we took the advantages of transgenic lines available for *Drosophila* to do further investigation, because we are lacking of genetic tools to do it on the *Locusta* wing to test whether and how *sal* regulates the expression of *ban. ban* transcription was monitored by *br-C12-lacZ*^35^ which was mainly expressed in the hinge/blade folds surrounding the wing pouch (Fig. 6A). Expression of *UAS-salm* in the medial region of the wing disc driven by *dpp-Gal4* apparently upregulated *ban* transcription level (arrows in Fig. 6B and B’). To confirm this result, clones were generated in the wing disc. Consistently, *ban* was upregulated in both medial regions and lateral region (Fig. 6C–C”). Therefore, *sal* promotes *ban* expression in the wing disc in a non-regional specific manner, unlike the manner of *omb*.

Previous studies have illustrated several models to explain how Dpp gradient controls a uniform cell proliferation rate in the wing disc^37^. Dpp restricts the transcription factor Brinker (Brk) domain to the lateral wing disc where suppresses the growth promoter *ban*^15^. While Brk activity is not essential for the medial wing disc growth^38^,^39^,^40^. Omb is a main mediator of Dpp signaling in the control of cell proliferation rate. Omb represses *ban* in medial regions, however, promotes it in lateral regions^20^. Distinct to *omb, sal* promotes *ban* expression in the whole wing disc (Fig. 6). Therefore, *sal* may mediate partial functions of Dpp in growth control. Other possibility is that *sal* may mediate the roles of other upstream factors such as Lines^41^, Wingless^42^, and Ubx in *Drosophila*^43^ as well as *Tribolium*^44^.

## Additional Information

**How to cite this article:** Wang, D. *et al. spalt* is functionally conserved in *Locusta* and *Drosophila* to promote wing growth. *Sci. Rep.* **7**, 44393; doi: 10.1038/srep44393 (2017).

**Publisher's note:** Springer Nature remains neutral with regard to jurisdictional claims in published maps and institutional affiliations.

## Supplementary Material

Supplementary Information
