# Supplementary material for: spalt is functionally conserved in Locusta and Drosophila to promote wing growth
Source: Sci Rep. 2017 Mar 16;7:44393. doi: 10.1038/srep44393 (PMC5353606; doi:10.1038/srep44393)
Supplement: Supplementary Information [file srep44393-s1.pdf]

spalt is functionally conserved in _Locusta_ and _Drosophila_ to promote wing growth

Dan Wang, Juanjuan Li, Suning Liu, Hang Zhou, Long Zhang, Wangpeng Shi, and Jie Shen*

Department of Entomology, China Agricultural University, Beijing100193, China

Supplementary Information

Sup Fig. 1 Lmsal cDNA and putative translation

Lmsal411 (1-2087 bases)

1 TGAGGTTTCGTTCTAGCTGATATTTTTAGAGGACGTATTTTAAATGACTGTTTTAACGATACCCGTGTTCTGAACCTAATTTTCGTTTTC
1 * G F V L A D I F R G R I L N D C F N D T R V L N L I F V F

91 TTCTGTTCCAGGTGAGTACCCAGTGTTGAATGCCTCTGCACCATGCAAGTCTTACCTGGTAAGTCGGAGCAAGCTTCGCCGTGTTCTGAA
31 F C S R * V P S V E C L C T M Q V L P G K S E Q A S P C S E

181 GTGGCCTGTTACCAGTTCAGTATTCGCTCTCGAAATCACGATAAAGCGGACTTTCGACTGCAGCGGCGCCTGCAATGCATCGCATCACCA
61 V A C Y Q F S I R S R N H D K A D F R L Q R R L Q C I A S P

271 AAGACTTCGGCGCTGGCCTTCGCCACGACACTGTCCGCGTGCGCCGACGAGGACGAGGCCAGCTCGTGCTCGGAGGCGGCAGTCTGTGAA
91 K T S A L A F A T T L S A C A D E D E A S S C S E A A V C E

361 GAAGCCGCCGACGTCGACGCCGATGCGGACGGAGACGTGGACGCGGACGGCGAACCAGACGCCGACTGCGACATGATGCTGCTCGACGAG
121 E A A D V D A D A D G D V D A D G E P D A D C D M M L L D E

451 CGCAACAACAACGAGGACGACGTAGAGCCCGAGGACGCCGTCTGCGCCGACGTGGCCGACGGAGTCGTCGGCTCCGGCGACGGAGACGCA
151 R N N N E D D V E P E D A V C A D V A D G V V G S G D G D A

541 GACACCGACGAGCCCGAGCCGGACGCCGAGCCCGAGGCGCTGACGTCCCCGGCGGCGGGCGTGGTGTCGGCGTTCGGGGCTGCGGTGGCG
181 D T D E P E P D A E P E A L T S P A A G V V S A F G A A V A

631 CCGTTCCCGCTGGCAGGGCACGTGACGCTGGAGGCGCTGCAGAACACCAAGGTGGCCGTGGCGCAGTTCGCCGCCACCGCCATGGCCGGC
211 P F P L A G H V T L E A L Q N T K V A V A Q F A A T A M A G

721 AACGCCGACAACGCCGCCGCGCTGCAGGAGCTCGCCGTGCTGCAGTCCACGCTCTTCACGCTGCAGCACCAACAGGTGATGCAACTATCG
241 N A D N A A A L Q E L A V L Q S T L F T L Q H Q Q V M Q L S

811 TTGATCCAGCAGCTGCAGCAGCAGCTACAGATCACACGCTGCCCCAAGGAGGCAGTGGCGCTCGCGAGTCCACCCCCGGTGCCTGCACCA
271 L I Q Q L Q Q Q L Q I T R C P K E A V A L A S P P P V P A P

901 GCAGCAGGACCTGCCTCCGCCCCCGATGCCAGCCCTCCGCCCCCACTGGCACTGCCCCTACCAGCCCAGCAGCAGCACCCATCGCTAGCG
301 A A G P A S A P D A S P P P P L A L P L P A Q Q Q H P S L A

991 CAGGTGCAGCAGTCGACACCTCCGAGCACGCAGTCACTACCCGCCTCTCCGCCTGCTGCCAAGCAGCAGCTTAAGGTGGCGGGGCCATCT

331 Q V Q Q S T P P S T Q S L P A S P P A A K Q Q L K V A G P S

1081 CCTCCACCACCACCTCCGCCCCCACAGGCAGTTCCACAGCAGCAGCAGCTCCACTCGCAGCTCCAGCCACAGCAGCAGCCAGCCCAGGTG

361 P P P P P P P P Q A V P Q Q Q Q L H S Q L Q P Q Q Q P A Q V

1171 CCCCAGCAGCTGCTGCCATGCTCCATCTCGTCATCACTGGCGGCATCTATCATTACCAACCCAGAGCCCCCACCACTCAATGAGCCCAAC

391 P Q Q L L P C S I S S S L A A S I I T N P E P P P L N E P N

1261 ACGTTGGAGATGTTGCAGCGGCGTGCACAAGAGGTACTAGAGAATGCCAGCCAGGGTCTGCTGGCCAACAACCTGGCCGATGAGCTGGCC

421 T L E M L Q R R A Q E V L E N A S Q G L L A N N L A D E L A

1351 TTCCGCAAGGGCGGTGCCGGCGGCAAGTCCAGCTCGCTCTCCCCATACGACTCCAAGTCGGGCCGGAACGAACCCTTCTTCAAGCACCGG

451 F R K G G A G G K S S S L S P Y D S K S G R N E P F F K H R

1441 TGTCGCTACTGTGGAAAGGTCTTCGGAAGTGACTCGGCACTGCAGATACACATCAGGTCGCACACGGGAGAGCGACCGTTCAAGTGCAAC

481 C R Y C G K V F G S D S A L Q I H I R S H T G E R P F K C N

1531 GTATGTGGTAGTCGATTCACCACCAAGGGCAATCTGAAGGTCCATTTCCAGAGGCACTCGGCCAAATTCCCACACATCAAAATGAACCCG

511 V C G S R F T T K G N L K V H F Q R H S A K F P H I K M N P

1621 AACCCAGTGCCAGAACACCTGGACAAGTATCACCCTCCACTGCTGGCACAACTGGGACAAAGCCTGTCACCGCACGGACAGCAGCCGACA

541 N P V P E H L D K Y H P P L L A Q L G Q S L S P H G Q Q P T

1711 CCACCTGTGCCACCAGTCCCACCCCCTCAGAGCCCATTCAGCTTCCCACTGTACAGACCTCCGCAGCCACCGCCTCCACCCCCACAGCAA

571 P P V P P V P P P Q S P F S F P L Y R P P Q P P P P P P Q Q

1801 CAGCAAGCGCAGCATCAGCAACAGCAGCAGCCGTCACAGCAACAGCAGCAAAAACCACAGCCACCACAGCTGCACTCACAGCCCCAGAGA

601 Q Q A Q H Q Q Q Q Q P S Q Q Q Q Q K P Q P P Q L H S Q P Q R

1891 CCGCAACTGCCTGGAACCGCTAATGGGAGCCTACCTCACAACTCACCTGGCTCAGATATACGACACGGTCCTCGTCCGAGGCAGGGACAC

631 P Q L P G T A N G S L P H N S P G S D I R H G P R P R Q G H

1981 GACATCGGACCGAGGATGCCTCACGGTGGCGGAAGCAGCTACGGTCCGCCCCGACCGATGGCTGCGGCCCCACCCCCTCCGCCCCCACCT

661 D I G P R M P H G G G S S Y G P P R P M A A A P P P P P P P

2071 GGCCAGCCCGGGGTCGG

691 G Q P G V

Lmsal468 (1-1073 bases)

```
1      GCAGCAACAACAACAACAACAACCGCGACCTCATGAACAGCAGCAGCAGCAGCAGCAGGATGCCAATGAGGAGACACCGTTTGTCAAACA
1       Q  Q  Q  Q  Q  Q  Q  P  R  P  H  E  Q  Q  Q  Q  Q  Q  Q  D  A  N  E  E  T  P  F  V  K  Q

91     GGAAGAGATGGATGACATTGGGTCAACGCAGAGGCGGCAAGATGATGACGAGTGTGAAGGTCACCTTAGTCCGAAGCGTGAACCACCAGA
31      E  E  M  D  D  I  G  S  T  Q  R  R  Q  D  D  D  E  C  E  G  H  L  S  P  K  R  E  P  P  E

181    GGAACCGGAGGATGTCGATATGTCTGCAAGGTTCCCATCATCTTCACCATATGATGACAGTCTGGACAGTAAATACAACAGCCAAGATGA
61      E  P  E  D  V  D  M  S  A  R  F  P  S  S  S  P  Y  D  D  S  L  D  S  K  Y  N  S  Q  D  E

271    GGAGAACAGCCTACAAGACCAGCCAGAGAATCTGTCCAGTAAAGGTGCGGGTGCCTCTAGTAGGCTCTCGCCACCAAGTTCAACGTCTTC
91      E  N  S  L  Q  D  Q  P  E  N  L  S  S  K  G  A  G  A  S  S  R  L  S  P  P  S  S  T  S  S

361    TGGCAGTGCAGTCGCCATAGACCCTGCTAAGGACCCAGCCATCTACACCAGTCTCCTACCACGCCCGGGAAGTAATGACAACTCGTGGGA
121     G  S  A  V  A  I  D  P  A  K  D  P  A  I  Y  T  S  L  L  P  R  P  G  S  N  D  N  S  W  E

451    GTCCCTCATTGAGATCACCAAAACATCGGAGACATCCAAGCTGCAGCAGCTTGTCGACAATATTGAGCACAAATTGACCGACCCAAACCA
151     S  L  I  E  I  T  K  T  S  E  T  S  K  L  Q  Q  L  V  D  N  I  E  H  K  L  T  D  P  N  Q

541    GTGTGTTATCTGCCACCGTGTCCTATCGTGCAAGAGTGCCCTGCAAATGCACTACCGTACGCATACTGGTGAACGGCCCTTCAAGTGCAA
181     C  V  I  C  H  R  V  L  S  C  K  S  A  L  Q  M  H  Y  R  T  H  T  G  E  R  P  F  K  C  K

631    GATATGTGGTCGTGCATTCACAACTAAGGGGAACCTCAAGACACACATGGGCGTGCACCGTGCCAAGCCGCCTGCACGAGTGCTGCACCA
211     I  C  G  R  A  F  T  T  K  G  N  L  K  T  H  M  G  V  H  R  A  K  P  P  A  R  V  L  H  Q

721    GTGCCCCGTATGCCATAAGAAGTTCACCAATGCACTGGTACTGCAGCAGCACATCAGGTTGCACACAGGAGAACCCACCGACCTGACCCC
241     C  P  V  C  H  K  K  F  T  N  A  L  V  L  Q  Q  H  I  R  L  H  T  G  E  P  T  D  L  T  P

811    AGAGCAGATACAAGCTGCAGAGGTGAAGGACTTTCCTGCAGCAGCCTCAGCTCCAGGGCCTGCCTTTCCTGGTCCAGTTTCGTTTCTGCA
271     E  Q  I  Q  A  A  E  V  K  D  F  P  A  A  A  S  A  P  G  P  A  F  P  G  P  V  S  F  L  Q

901    GGCATTCCCACCATTGCCACCACCTGGCATTCACCTTCCACCTGCACAGCACCACACAAAGCTATCAGCAGGATCCACACATGGGGATTT
301     A  F  P  P  L  P  P  P  G  I  H  L  P  P  A  Q  H  H  T  K  L  S  A  G  S  T  H  G  D  F

991    TAAGGAAGAAAAGCCTGACTATCCTGACGATGATAATTCAAGTAGCAGTGGGGGTGGAGGGCAGCAGCAGCAGCAACAACAGC
331     K  E  E  K  P  D  Y  P  D  D  D  N  S  S  S  S  G  G  G  G  Q  Q  Q  Q  Q  Q
```
